# Untargeted Metabolomics Investigation on Selenite Reduction to Elemental Selenium by *Bacillus mycoides* SeITE01

**DOI:** 10.3389/fmicb.2021.711000

**Published:** 2021-09-16

**Authors:** Greta Baggio, Ryan A. Groves, Roberto Chignola, Elena Piacenza, Alessandro Presentato, Ian A. Lewis, Silvia Lampis, Giovanni Vallini, Raymond J. Turner

**Affiliations:** ^1^Department of Biotechnology, University of Verona, Verona, Italy; ^2^Department of Biological Sciences, University of Calgary, Calgary, AB, Canada; ^3^Department of Biological, Chemical and Pharmaceutical Sciences and Technologies (STEBICEF), University of Palermo, Palermo, Italy

**Keywords:** *Bacillus mycoides* SeITE01, selenite, selenium nanoparticles, signaling molecules, time course, untargeted metabolomics

## Abstract

*Bacillus mycoides* SeITE01 is an environmental isolate that transforms the oxyanion selenite (${\mathrm{SeO}}_{3}^{2-}$) into the less bioavailable elemental selenium (Se^0^) forming biogenic selenium nanoparticles (Bio-SeNPs). In the present study, the reduction of sodium selenite (Na_2_SeO_3_) by SeITE01 strain and the effect of ${\mathrm{SeO}}_{3}^{2-}$ exposure on the bacterial cells was examined through untargeted metabolomics. A time-course approach was used to monitor both cell pellet and cell free spent medium (referred as intracellular and extracellular, respectively) metabolites in SeITE01 cells treated or not with ${\mathrm{SeO}}_{3}^{2-}$. The results show substantial biochemical changes in SeITE01 cells when exposed to ${\mathrm{SeO}}_{3}^{2-}$. The initial uptake of ${\mathrm{SeO}}_{3}^{2-}$ by SeITE01 cells (3h after inoculation) shows both an increase in intracellular levels of 4-hydroxybenzoate and indole-3-acetic acid, and an extracellular accumulation of guanosine, which are metabolites involved in general stress response adapting strategies. Proactive and defensive mechanisms against ${\mathrm{SeO}}_{3}^{2-}$ are observed between the end of lag (12h) and beginning of exponential (18h) phases. Glutathione and N-acetyl-L-cysteine are thiol compounds that would be mainly involved in Painter-type reaction for the reduction and detoxification of ${\mathrm{SeO}}_{3}^{2-}$ to Se^0^. In these growth stages, thiol metabolites perform a dual role, both acting against the toxic and harmful presence of the oxyanion and as substrate or reducing sources to scavenge ROS production. Moreover, detection of the amino acids L-threonine and ornithine suggests changes in membrane lipids. Starting from stationary phase (24 and 48h), metabolites related to the formation and release of SeNPs in the extracellular environment begin to be observed. 5-hydroxyindole acetate, D-[+]-glucosamine, 4-methyl-2-oxo pentanoic acid, and ethanolamine phosphate may represent signaling strategies following SeNPs release from the cytoplasmic compartment, with consequent damage to SeITE01 cell membranes. This is also accompanied by intracellular accumulation of trans-4-hydroxyproline and L-proline, which likely represent osmoprotectant activity. The identification of these metabolites suggests the activation of signaling strategies that would protect the bacterial cells from ${\mathrm{SeO}}_{3}^{2-}$ toxicity while it is converting into SeNPs.

## Introduction

*Bacillus mycoides* SeITE01 is an aerobic rod-shaped endospore-forming Gram-positive bacterium belonging to the *Firmicutes* phylum that was isolated from the rhizosphere of the Se-hyperaccumulator plant *Astragalus bisulcatus* grown in seleniferous soils (Vallini et al., 2005). SeITE01 strain shows the ability to withstand high concentrations of ${\mathrm{SeO}}_{3}^{2-}$ (up to 25mM), reducing this oxyanion into the insoluble and less bioavailable elemental selenium (Se^0^) with the generation of biogenic selenium nanoparticles (Bio-SeNPs) (Lampis et al., 2014; Piacenza et al., 2019; Bulgarini et al., 2020). In recent years, SeNPs have been the subject of great interest due to their attractive characteristics. These nanostructures can be used in both the technological and industrial fields thank to their special physical features, such as semiconducting, photoelectric, and X-ray-sensing properties (Wadhwani et al., 2016). At the same time, it was shown that Bio-SeNPs can exert an efficient and high antibacterial activity against human pathogens, such as *Escherichia coli*, *Pseudomonas aeruginosa*, and *Staphylococcus aureus* (Zonaro et al., 2015; Cremonini et al., 2016; Piacenza et al., 2017) and inhibit the formation of bacterial biofilms on medical-hospital devices (Sonkusre and Cameotra, 2015).

While there has been progress in establishing mechanisms of metal ion toxicity to bacteria (Lemire et al., 2013), a complete understanding of all the biochemical processes from various metal(loid) ion exposures is far from complete. Various “-omics” approaches can be employed to help fill the knowledge gaps. The use of metabolomics to elucidate cell responses to metal exposure began about a decade ago (Booth et al., 2011a). Metabolomics are a relatively recent addition to the “-omics” toolbox with the advent of improvements in technologies, allowing it to be a growing discipline in the field of biological systems (Dettmer and Hammock, 2004). Complementing other “-omics”, this powerful tool studies the turnover of biochemicals in living cells (Villas-Bôas et al., 2007; Baidoo et al., 2012). Since metabolites are subjected to continuous turnover, their levels and distributions can have enormous spatial and temporal variability. Some metabolites can accumulate within cells and be highly abundant (in the range of mM), while others may be quickly transformed and/or consumed and be present only in small traces (in the order of pM; Warwick and Ellis, 2005; Baidoo et al., 2012). Consequently, the metabolite concentration and flux through various biochemical networks can provide integrative information on the physiological state and response to stress of a living organism. Several studies have been conducted to examine the metabolic responses of different bacterial strains exposed to metals, such as cadmium (Cd), copper (Cu), aluminum (Al), gallium (Ga) (Beriault et al., 2006; Lemire et al., 2008; Booth et al., 2011a; Zhai et al., 2018), and the metalloid oxyanion tellurite (${\mathrm{TeO}}_{3}^{2-}$) (Tremaroli et al., 2009). These investigations analyzed the relationship between the metal stress and the bacterial behavior comparing free swimming planktonic populations with surface-attached biofilms (Booth et al., 2011b) or wild-type cells with mutants (Tremaroli et al., 2009). These studies have revealed multiple effects exerted by metals into bacterial cells in terms of biochemical changes and reconfiguration of cell metabolism (Beriault et al., 2006; Lemire et al., 2008; Tremaroli et al., 2009; Booth et al., 2011b; Zhai et al., 2018). Although the results obtained have demonstrated the ability to distinguish and describe the diverse strain phenotypes in response to the exposure of different metals, these metabolomics analyses used an end-point approach. In fact, most attention has been given to the quantitative end-point study of metal(loid) resistance and tolerance of different microbial strains (Beriault et al., 2006; Lemire et al., 2008; Tremaroli et al., 2009; Booth et al., 2011b; Zhai et al., 2018) and less work has asked how the different metal(loid) ions exert their toxic effects (Lemire et al., 2013).

In the present study, SeITE01 cells growing in the presence of ${\mathrm{SeO}}_{3}^{2-}$ were evaluated at different stages of growth. Liquid Chromatography Mass Spectrometry (LC-MS) (Haggarty and Burgess, 2017; Kamphorst and Lewis, 2017; Kamal and Sharad, 2018) was used to find metabolite changes in *B. mycoides* SeITE01 cultures treated or not with Na_2_SeO_3_ in order to evaluate the effect of ${\mathrm{SeO}}_{3}^{2-}$ oxyanion on bacterial cells while they are reducing it with the generation of Bio-SeNPs along a 48-h time course.

## Materials and Methods

### Culture Media, Chemicals, and Solutions

Oxoid™ Nutrient Broth (NB) and Oxoid™ Agar Bacteriological were provided by Thermo Fisher Scientific™ (Ontario, Canada). Chemicals at the analytical grade were purchased from Merck KGaA (Ontario, Canada). Na_2_SeO_3_ was prepared as a 500mM stock solution in deionized water and sterilized by filtration (0.2μm; Sarstedt Inc., Fisher Scientific™). Phosphate-buffered saline (PBS) solution was prepared at the final concentration of 100mM and pH=7.4, while methanol and double distilled water (MetOH-ddH_2_O) have been mixed in a v/v ratio 1:1.

### Bacterial Strain and Growth Conditions

*Bacillus mycoides* SeITE01 was aerobically pre-cultured for 24h at 27°C on an orbital shaker (150rpm; G10 Gyrotory Shaker, New Brunswick Scientific CO., Inc.) in 50-ml Erlenmeyer flasks containing 20ml of the rich medium NB. Na_2_SeO_3_ stock solution (500mM) was added to the culture media at the final concentration of 2.0mM. Bacterial growth was carried out in 250-ml Erlenmeyer flasks containing 100ml of NB supplied or not with 2.0mM ${\mathrm{SeO}}_{3}^{2-}$ (namely, ${\mathrm{SeO}}_{3}^{2-}$-treated and untreated, respectively) and inoculated with pre-cultured cells at an optical density (OD_600_; Hitachi U-2000 Spectrophotometer) of 0.01. All microbiological experiments were conducted in biological triplicates (*n*=3).

### Evaluation of Bacterial Growth and $SeO_{3}^{2-}$ Depletion

Growth profiles of SeITE01 cultured in presence or absence of Na_2_SeO_3_ were evaluated at different time points, namely, after 3, 6, 9, 12, 18, 24, and 48h of incubation. Growth was monitored by Colony Forming Units (CFU) counting on Nutrient Agar plates and reported as the logarithm of the CFU per milliliter (Log_10_ CFU/ml) of culture with standard deviation (SD). ${\mathrm{SeO}}_{3}^{2-}$ depletion in the medium was determined spectrophotometrically (Varian Cary® 50 Bio UV–Vis) as previously described (Kessi et al., 1999; Lampis et al., 2014). ${\mathrm{SeO}}_{3}^{2-}$ concentration was evaluated by measuring the absorbance of the organic phase at 377nm of the Se-2, 3-diaminonaphthalene complex in cyclohexane, using a 1-cm path length quartz cuvette (Hellma® Analytics) against a calibration curve (*R*^2^=0.9876) calculated as average value (*n*=3) and constructed by using 0, 50, 100, 150, and 200nmol of ${\mathrm{SeO}}_{3}^{2-}$ dissolved in liquid NB medium.

### TEM Analysis

The imaging of SeITE01 cells was performed using a Hitachi H-7650 120kV transmission electron microscope (TEM) as described elsewhere (Piacenza et al., 2018). Aliquots (500μl) of bacterial cultures either supplied or not with ${\mathrm{SeO}}_{3}^{2-}$ were recovered at the same incubation times chosen for the metabolomics analysis (3, 12, 18, 24, and 48h) and centrifuged at 16,000*g* for 10min at 4°C. The obtained cell pellets were diluted in 10μl of ddH_2_O to reach a final CFU/ml value of 4×10^4^, deposited on CF300-Cu-Carbon Film Copper grids, and air dried for 24-h prior to their observation.

### Metabolite Extraction

Samples from untreated and ${\mathrm{SeO}}_{3}^{2-}$-treated cultures were collected at 3, 12, 18, 24, and 48h of bacterial growth. Preparation of SeITE01 intracellular samples for both experimental conditions was always started from the same number of CFU equal to 2×10^6^/ml. Cell pellets were centrifuged at 16,000*g* for 10min at 4°C, washed once with cold PBS solution, and immediately stored at −80°C until use. The metabolite extraction protocol involved taking and re-suspending the cell pellets in 100μl of a pre-cooled (−20°C) mixture of MetOH-ddH_2_O, followed by 1min of vortexing and 10min of centrifugation at 16,000*g* at 4°C. 80μl of the suspensions was then transferred into clean glass vials and analyzed. A different approach was adopted for the extracellular samples. 500μl of each sample was collected, centrifuged at 16,000*g* for 10min at 4°C to remove residual bacterial cells, transferred in new and clean tubes, and stored at −80°C until use. 50μl of the chilled supernatants was then added to 950μl of the cold MetOH-ddH_2_O in order to reach a dilution equal to 1:20. The suspensions were vortexed for 1min, centrifuged again at 16,000*g* for 10min at 4°C, and ultimately, 800μl was analyzed by LC-MS.

### LC-MS Acquisition

Metabolites present in the extracts were separated using ultra high-performance liquid chromatography on a Thermo Scientific Vanquish Horizon UHPLC system. A binary mixture of 20mM ammonium formate at pH 3.0 in water (Solvent A) and 0.1% (v/v) formic acid in acetonitrile (Solvent B) was used in conjunction with a Syncronis™ HILIC LC column (100mm×2.1mm×2.1μm; Thermo Scientific). The following analytical gradient was used (with respect to percentage of solvent B) to achieve chromatographic separation: 100% from 0 to 2min; 100 to 80% from 2 to 7min; 80 to 5% from 7 to 10min; 5% from 10 to 12min; 5 to 100% from 12 to 13min; and 100% from 13 to 15min. High-resolution mass spectral data were obtained on a Thermo Scientific Q-Exactive™ HF Hybrid Quadrupole-Orbitrap mass spectrometer coupled to a Thermo Scientific Ion Max-S API Source. Data were acquired in negative ion full-scan mode from 50 to 750 mass to charge ratio (m/z) at 240,000 resolution with automatic gain control target of 3×10^6^ ions and a maximum injection time of 200ms. Identification and relative quantification of both intracellular and extracellular metabolites were carried out with the open source software Metabolomic Analysis and Visualization ENgine (MAVEN; Melamud et al., 2010). Metabolite peak assignments were determined by matching the previously established m/z and retention time (RT) of authentic standards with observed metabolite signals.

### Statistical Analysis

Graphic representation of the clustered heat maps of raw data for the intracellular and extracellular dataset was obtained with R-3.3.3 software.^1^

Identification of metabolites whose concentration varied significantly between treatment conditions from the analysis of their temporal changes was performed with two advanced statistical approaches. They were carried out with the open source platform for statistical computing and graphics R (version 3.6.0) run under the free integrated development environment RStudio (version 1.0.153).^2^ The first method exploited multivariate empirical Bayes statistics to test the null hypothesis that the two expected profiles were the same. A T^2^ statistics equivalent to the two-sample Hotelling T^2^ statistics have been derived by considering a degree of moderation of the variance-covariance matrices toward a common matrix which retained the temporal correlation structure of the data (Tai and Speed, 2006). A ranking of metabolites’ profiles that varied at most in time between the cultures supplemented and not with ${\mathrm{SeO}}_{3}^{2-}$ was then computed. The top 10% of compounds in this ranking were considered for further analyses. The full algorithms are available in the time-course R package (Tai, 2019). The second approach was instead developed in-house. Data analysis started by considering each metabolite a multidimensional vector of time-course data samples, exploiting Principal Component Analysis (PCA) to identify the temporal dimensions where the samples varied at most. The vectors deriving from the Euclidean distances between untreated and ${\mathrm{SeO}}_{3}^{2-}$-treated samples were expected to follow a Rayleigh distribution that therefore defined the null hypothesis for statistical comparisons (fitdistrplus package, Delignette-Muller and Dutang, 2015). A conservative significance threshold was set at *p*<10^−4^ for the rejection of the null hypothesis. Ultimately, metabolites identified by these statistical approaches were compared using Venn diagrams and used for the reconstruction of the final clustered heat maps. One further concern was that the raw data showed narrow peaked distributions with very long tails. To prevent a few metabolites from dominating the statistical comparisons because of high leverage, bestNormalize package was used to search for the best normalization procedure (Peterson, 2019). Intracellular metabolites were then normalized applying the BoxCox parametric transform (Zar, 2010) implemented in the geoR package (Ribeiro and Diggle, 2018), whereas for extracellular metabolites were used the Ordered Quantile normalization transformation (Bartlett, 1947) provided by the orderNorm function implemented in the bestNormalize package.

## Results

### Growth Under $SeO_{3}^{2-}$ Exposure

**Figure 1:**
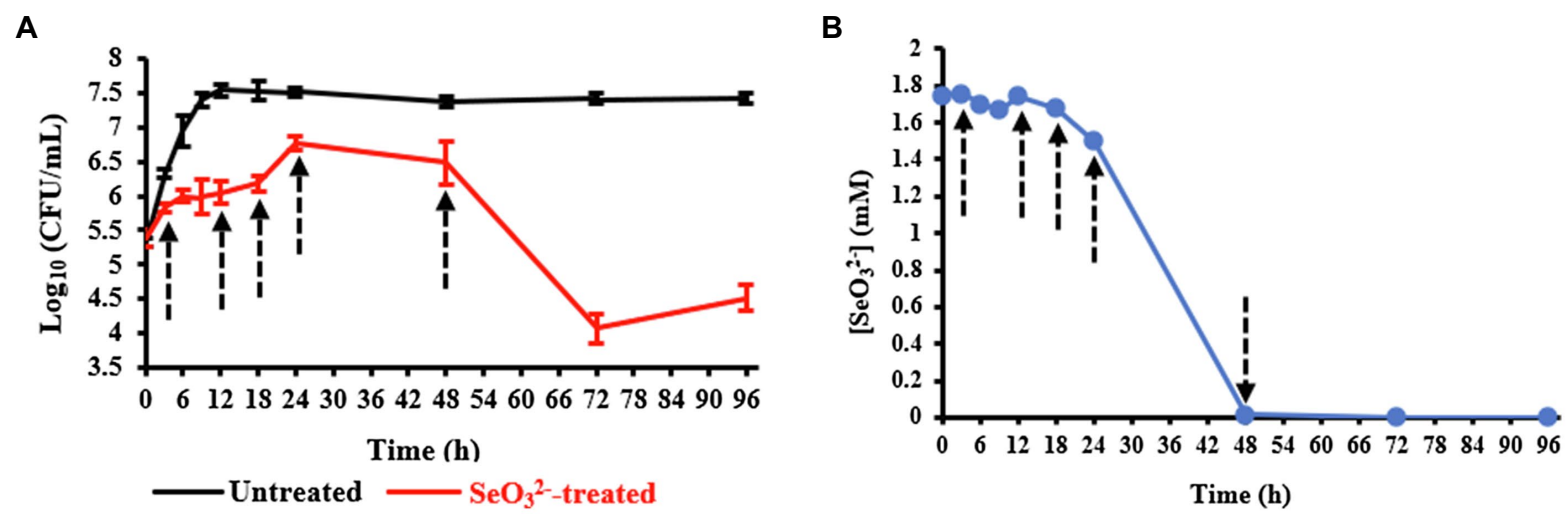
Panel **(A)** shows the growth profiles of *Bacillus mycoides* SeITE01 supplied or not with 2.0mM Na_2_SeO_3_. Panel **(B)** displays ${\mathrm{SeO}}_{3}^{2-}$ consumption by *B. mycoides* SeITE01 culture over time. Dotted arrows indicate the time points chosen for metabolomics investigation.

Evaluation of the growth of *B. mycoides* SeITE01 exposed or not to 2.0mM Na_2_SeO_3_ is shown in Figure 1A. Bacterial cultures in absence of ${\mathrm{SeO}}_{3}^{2-}$ displayed a short lag phase of 3h, while stationary phase was reached after about 18h. On the other hand, the growth dynamics and final cells yield were negatively affected by the presence of ${\mathrm{SeO}}_{3}^{2-}$. An extended lag phase between 6h and 12h was observed, and both exponential and stationary phases were delayed to around 18h and 24h, respectively. Moreover, in the ${\mathrm{SeO}}_{3}^{2-}$ exposed samples, the death phase was reached after 48h, while in the untreated ones, an extended stationary phase up to 96h was observed. Data description is congruent with that previously presented in Vallini et al. (2005) and Lampis et al. (2014).

Upon evaluating the ${\mathrm{SeO}}_{3}^{2-}$ levels in the spent medium with time, ${\mathrm{SeO}}_{3}^{2-}$ oxyanion initially added was primarily depleted by cells after the stationary phase was reached (24-h time point) as shown in Figure 1B. Data obtained from both the growth curves and ${\mathrm{SeO}}_{3}^{2-}$ consumption allowed us to identify specific time points to be used for metabolomics investigation in order to capture relevant metabolic changes experienced by SeITE01 cells in ${\mathrm{SeO}}_{3}^{2-}$-treated conditions and during the oxyanion bio-reduction process.

### Cells Morphology Analysis in Presence of $SeO_{3}^{2-}$ and Bio-SeNPs Detection

Temporal evolution of the morphology of untreated and${\mathrm{SeO}}_{3}^{2-}$treated SeITE01 cells was investigated by TEM microscopy (Supplementary Figures 1 and 2). Untreated cells revealed expected development during the entire time course (Supplementary Figures 1A–E), exhibiting a rod-shaped morphology, which is typical of *Bacillus* sp. (Lampis et al., 2014). Growth in presence of ${\mathrm{SeO}}_{3}^{2-}$ (Supplementary Figure 2) induced, instead, some changes in cell morphology, especially in the early stages of growth (Supplementary Figures 2A–C). The typical rod-shaped morphology was reached by stationary phase (24h). At this time point, it was possible to observe nanostructures (spherical black or dark gray spots due to their electron dense nature) in both intracellular and extracellular space (Supplementary Figure 2D), which were not detected in untreated cell samples collected at the same time point, and that can be ascribed to SeNPs. By 48h, a considerable increase in the number of nanoparticles was found outside the bacterial cells (Supplementary Figure 2E). The appearance of Bio-SeNPs correlated with the depletion of ${\mathrm{SeO}}_{3}^{2-}$ observed between 24 and 48h as shown in Figure 1B.

### Metabolomics Investigation

**Figure 2:**
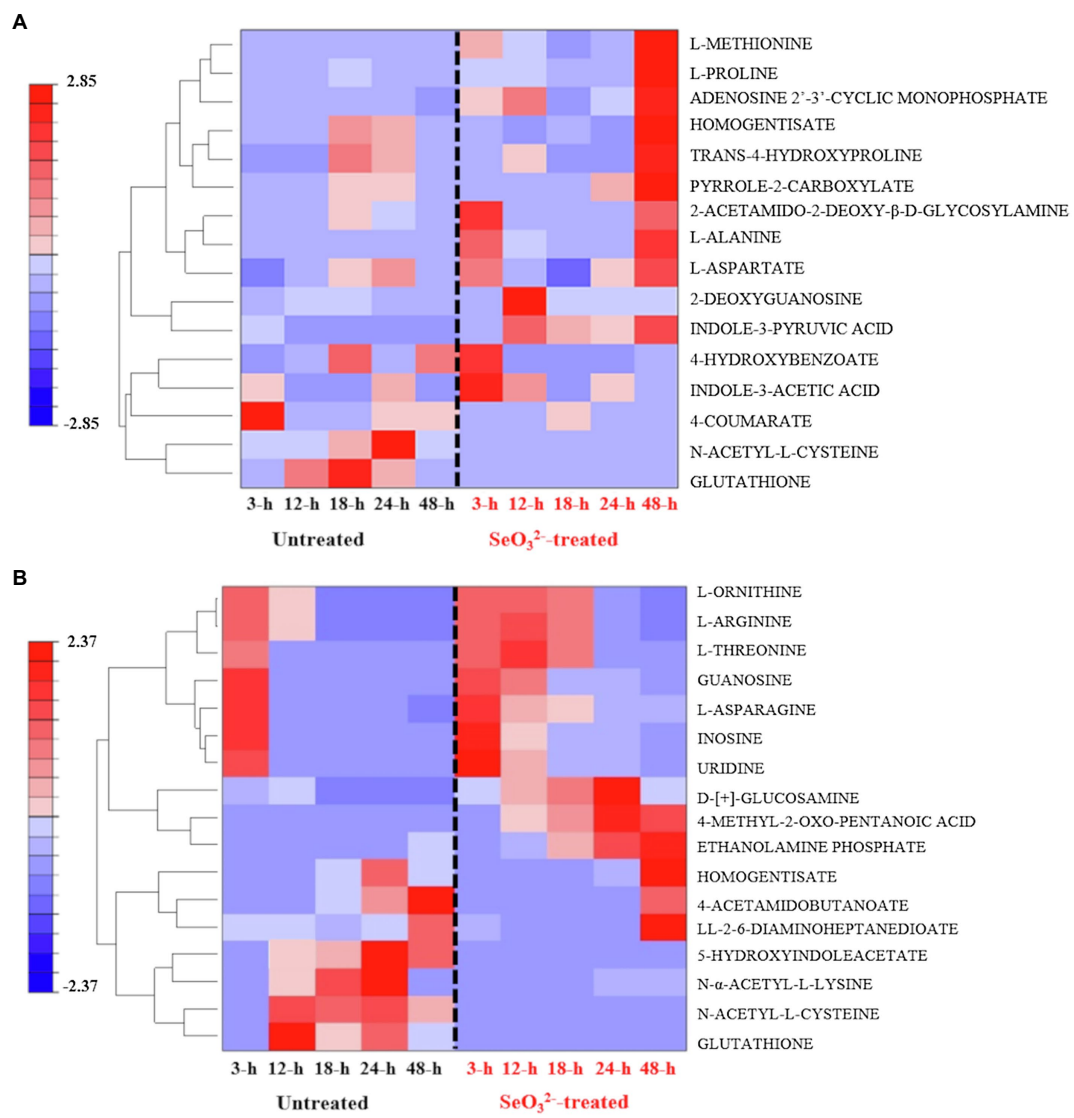
Clustered heat maps of the most statistically relevant intracellular **(A)** and extracellular **(B)** metabolites of *B. mycoides* SeITE01 identified by the two statistical approaches.

Our experiments were outlined to study the change of the biochemical state of SeITE01 cells at different points along the growth curve, namely, at beginning (3h) and end (12h) of lag phase, at early (18h) and late (24h) exponential phase and, finally, once they had reached stationary phase (48h) (Figure 1). Following metabolomic analysis, 125 compounds associated with the bacterial cell pellets (intracellular metabolites; >Supplementary Figure 3A) and 124 recurring in the cell free spent culture medium (extracellular metabolites; Supplementary Figure 3B) were initially identified. Graphical representations of the clustered heat maps underlined the complexity of the data. To identify significant variations in bacterial strain SeITE01 metabolism associated with ${\mathrm{SeO}}_{3}^{2-}$-exposure during the time course, data were analyzed by two robust statistical approaches: the combination of PCA with the Squared Euclidean Distance (Supplementary Figure 4, Supplementary Table 1) and the integration of Bayesian Inference with the T^2^ statistics (Supplementary Table 2). These analyses provided two lists of 16 and 19 statistically relevant metabolites from the intracellular and extracellular samples, respectively. Data were then compared through Venn diagrams to graphically identify metabolites in common with the two statistical approaches (Supplementary Figure 5) and used for the reconstruction of the final clustered heat maps (Figure 2). The two categories of samples showed distinct differences in both their trends during the time course and the metabolic pathways they are involved in.

### Intracellular Metabolites

Two key temporal responses can be observed within intracellular compounds of ${\mathrm{SeO}}_{3}^{2-}$-treated cells: an early (3h) and a late response (48h). Metabolites associated with the first response were 4-hydroxybenzoate (4-HB) and indole-3-acetic acid (IAA), while L-proline and trans-4-hydroxyproline belong to the second one. In the case of untreated bacterial cells, it was not possible to identify families of metabolites showing a definitive temporal trend. However, attention must mostly be paid to glutathione (GSH) and N-acetyl-L-cysteine (NAC), which are lacking in ${\mathrm{SeO}}_{3}^{2-}$-treated cells (Figure 2A).

### Extracellular Metabolites

Distribution of metabolites belonging to the extracellular dataset showed a characteristic behavior pattern of metabolites consumed or produced (Figure 2B). Compounds in the lower part of the clustered heat map displayed an identical temporal evolution between the two treatments (i.e., L-serine, L-aspartate, and 5-oxo-D-proline), while in the upper portion, differences are recognizable with some metabolites present only in the untreated samples (i.e., N-α-acetyl-L-lysine and succinate), while others are observed only in ${\mathrm{SeO}}_{3}^{2-}$-treated cultures (i.e., mono methyl glutarate, 3-dehydroshikimate, and D-ribose). Analysis of the most significant metabolites detected under ${\mathrm{SeO}}_{3}^{2-}$ exposure underlined the presence of four main classes of macromolecules: thiol redox and signaling molecules [e.g., GSH, NAC, and 5-hydroxyindoleacetate (5-HIAA)]; purine derivative, such as guanosine; amino acids (e.g., L-threonine and L-ornithine) and amino compounds (e.g., ethanolamine phosphate and D-[+]-glucosamine); and α-keto acid as 4 methyl-2-oxo pentanoic acid.

## Discussion

**Figure 3:**
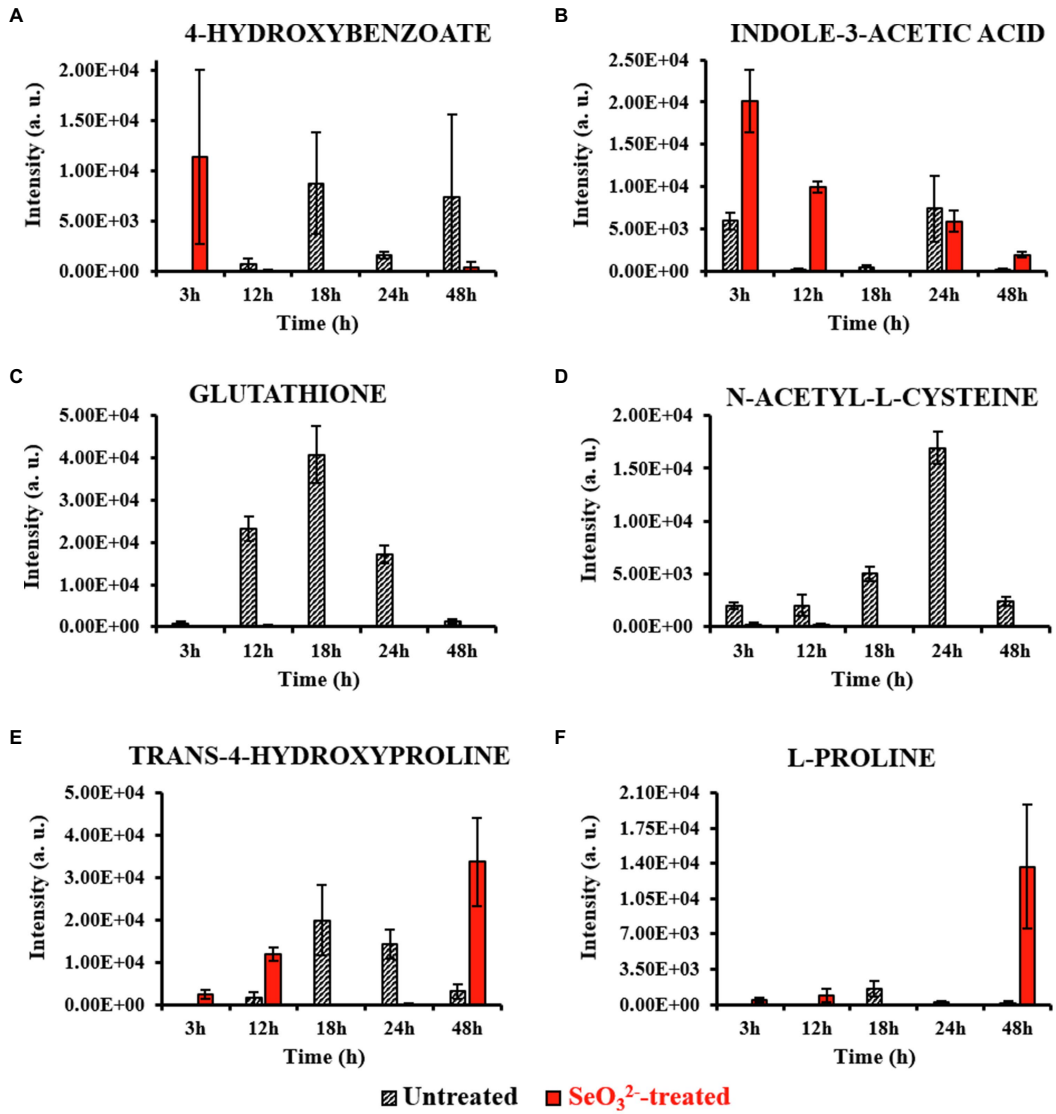
Most relevant intracellular metabolites. This figure shows the intensity trends of metabolites from both untreated and ${\mathrm{SeO}}_{3}^{2-}$-treated cells during the time course. Panels **(A,B)** show metabolites present in the earliest stage of growth (3h). Panels **(C,D)** show metabolites accumulated during the end of lag (12h) and beginning of exponential (18h) phases. Panels **(E,F)** show metabolites present late in stationary phase (48h) and during Bio-SeNPs release.

**Figure 4:**
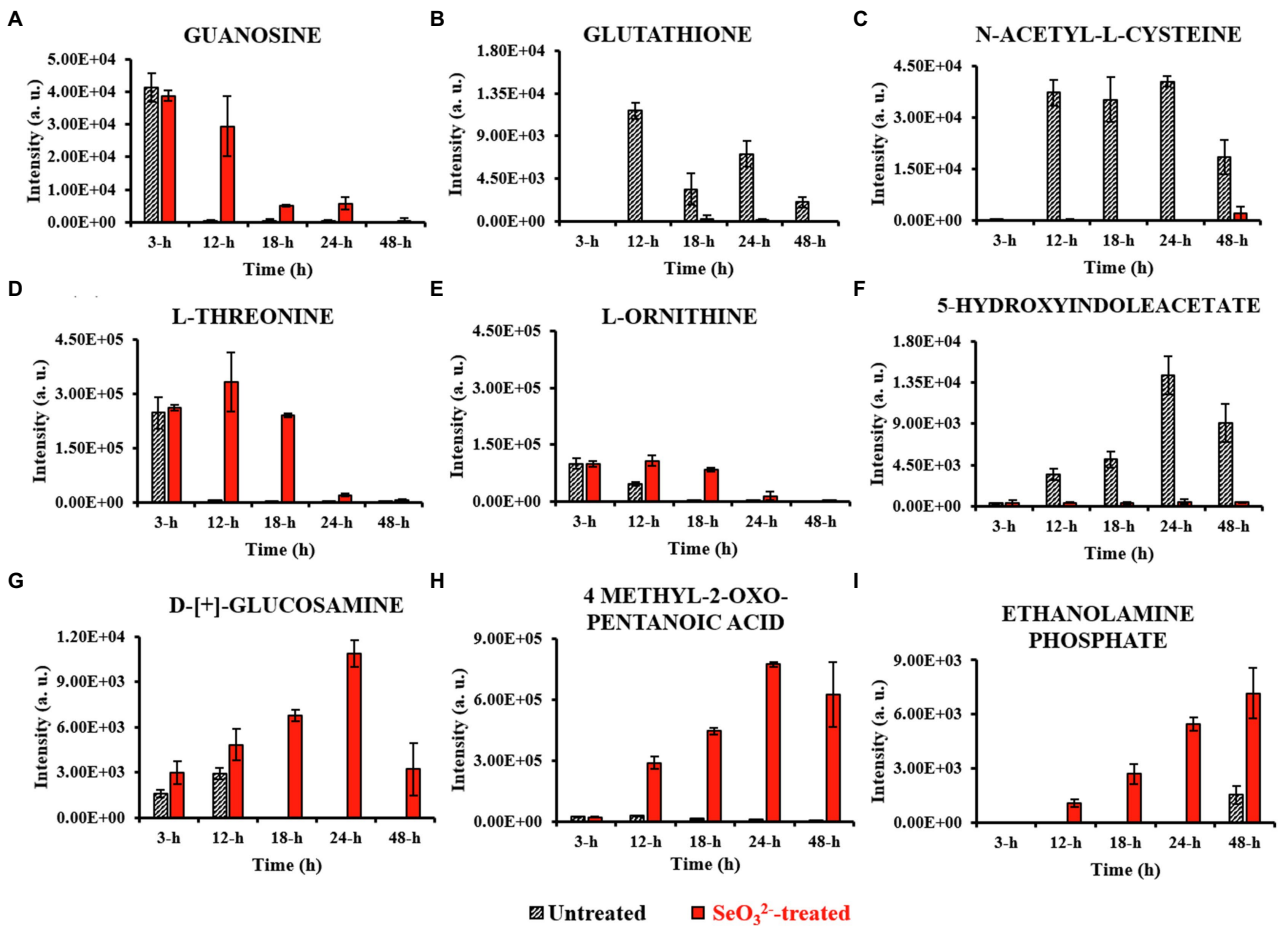
Most relevant extracellular metabolites. This figure shows the intensity trends of metabolites from both untreated and ${\mathrm{SeO}}_{3}^{2-}$-treated cells during the time course. Panel **(A)** shows metabolite present in the earliest stage of growth (3h). Panels **(B–E)** show metabolites extruded during lag (12h) and exponential (18h) stages. Panels **(F–H)** represent metabolites detected during stationary stage (24h), while panel **(I)** describes metabolite secreted in late stationary phase (48h).

Metabolomics can be considered a powerful tool for understanding and providing clues toward hypothesis for describing a given phenotype. Nevertheless, it is very sensitive to experimental design, sample preparation, statistical data analysis, and interpretation. Here, our statistical evaluation of both intracellular and extracellular datasets was complex. Cell culturing in rich NB medium would lead to a variety of metabolic pathways active, while the use of three different variables (untreated and ${\mathrm{SeO}}_{3}^{2-}$-treated cultures, and time) prevented an ease use of classic statistical approaches so far applied for metabolomics studies. The challenge was to evaluate not only a possible difference in metabolites between ${\mathrm{SeO}}_{3}^{2-}$-treated and untreated cultures, but also to see how this difference varies over the time course, possibly recognizing metabolites involved in ${\mathrm{SeO}}_{3}^{2-}$ bio-reduction process. The analysis of clustered heat maps deriving from the statistical processing of datasets (Figure 2) together with the graphic representation of the statistically relevant metabolites corresponding to different growth states allowed for the identification of the biomolecules that changed in the cell pellets (intracellular metabolites; Figure 3) and cell free spent medium (extracellular metabolites; Figure 4) samples. It is important to mention that interpretation of extracellular metabolites and identification of a possible relationship between these compounds and ${\mathrm{SeO}}_{3}^{2-}$-oxyanion effect requires considerably more caution than the elucidation of the intracellular pool. Extracellular compounds, in fact, can be the result of selective nutrient import, active efflux of metabolites from the cytoplasm, cell membrane leakage due to osmotic stress, or cell death leading to complete release of all cell constituents. Below follows the time course of the experiment of growth of SelTE01 under selenite exposure.

Lag phase of growth shows the initial uptake of ${\mathrm{SeO}}_{3}^{2-}$ by SeITE01 cells can be traced back to the earliest stage of growth, the 3-h time point. The oxyanion’s transport into cellular compartments is accompanied by a change in cell morphology (Supplementary Figure 2A), an increase in intracellular levels of 4-HB and IAA (Figures 3A,B), and the extracellular accumulation of guanosine (Figure 4A). 4-HB is a precursor of the primary electron transport chain carrier ubiquinone (Q) also involved in gene regulation and oxygen radical scavenging (Søballe and Poole, 1999). Its accumulation in ${\mathrm{SeO}}_{3}^{2-}$-treated cells suggests an oxidative stress role induced by ${\mathrm{SeO}}_{3}^{2-}$, acting as antioxidant molecule and ROS scavenger. Moreover, Sévin and Sauer (2014) have demonstrated that the intracellular accumulation of ubiquinone in *E. coli* can elicit osmotic-stress tolerance through the modification of cell membrane composition. IAA is an ubiquitous signaling molecule responsive to different stress conditions (Somers et al., 2005; Bianco et al., 2006; Zarkan et al., 2020). Finally, we see guanosine, which can play regulatory roles in stress response, biofilm formation, and cellular damage protection (Cornforth and Foster, 2013; Rowlett et al., 2017; Bange and Bedrunka, 2020). Thus, the observations at this growth phase suggest the cells elucidating general stress response adapting to the ${\mathrm{SeO}}_{3}^{2-}$ loaded environment.

From the end of the lag (12h) and exponential (18h) growth phases under selenite exposure, we see ${\mathrm{SeO}}_{3}^{2-}$ uptake but no conversion to Se^0^ (Figure 1B), and cells continue to present structural malformation (Supplementary Figures 2B,C). However, the detection of reductive thiol (RSH) compounds only in untreated samples and their total absence in the exposed ones allows us to hypothesize that ${\mathrm{SeO}}_{3}^{2-}$ has started reactions with these types of molecules, as expected. GSH (Figures 3C, 4B) and NAC (Figures 3D, 4C) were found at high levels both intracellularly and extracellularly for untreated samples, yet essentially absent from ${\mathrm{SeO}}_{3}^{2-}$ exposed ones. It is recognized that GSH and related thiol compounds are notoriously challenging to accurately quantify (Lu et al., 2017). Besides, it is known that RSH molecules are important for chalcogen chemistry through involvement in Painter reduction reactions of ${\mathrm{SeO}}_{3}^{2-}$ to Se^0^. In this way, the total absence of GSH and NAC in ${\mathrm{SeO}}_{3}^{2-}$-treated samples throughout the entire time course supports the literature observations that these metabolites are rapidly consumed (Painter, 1941; Ganther, 1971; Kessi et al., 1999; Kessi and Hanselmann, 2004; Kessi, 2006) and they may be involved in proactive toxicity. Furthermore, they act as a substrate or reducing sources to scavenge ROS produced as a consequence of ${\mathrm{SeO}}_{3}^{2-}$ reactions and cell damage, playing a defensive role (Turner et al., 1998). Extracellularly, we see the amino acids L-threonine (Thr) and ornithine (Orn) changing (Figures 4D,E). Orn is of interest as it plays numerous roles in cells as a biosynthetic precursor of arginine linked to the urea cycle (Stalon et al., 1987; Reitzer, 2009). It is also a key to the biosynthesis of polyamines, a class of compounds involved in a variety of cellular processes, such as gene expression, cell growth, survival, and stress response (Gevrekci, 2017). Additionally, Orn can be used for the synthesis of phosphorus-free ornithine lipids, which are alternative membrane lipids activated under stress and widespread among eubacteria (Sohlenkamp and Geiger, 2016; Sohlenkamp, 2019). Combining these growth phase observations, we can postulate that ${\mathrm{SeO}}_{3}^{2-}$ is reacting with RSH molecules, and subsequent stress response may be in part dealt with changes in membrane lipids.

Stationary phase (24h) marks the beginning of the SeNPs formation and extracellularly accumulation and the cells adapting a normal rod-shape morphology (Supplementary Figure 2D). 5-HIAA is an indole derivative with roles in virulence, cell cycle regulation, acid, pH and heat resistance, and a signaling molecule in biofilm formation (Hu et al., 2010; Lee and Lee, 2010; Zarkan et al., 2020). Cellular survival in this stage of growth is deeply linked to both energy metabolism and stress resistance (Wang et al., 2001; Hu et al., 2010; Gaimster et al., 2014; Zarkan et al., 2020). D-[+]-glucosamine (GlcN) and 4-methyl-2-oxo pentanoic acid are observed, increasing up to 24h. GlcN is a non-acetylated amino sugar whose acetylated form is one of the two components of peptidoglycan (Vollmer et al., 2008). In other species of *Bacillus*, the presence of this metabolite followed the activation of defense responses against external agents (Psylinakis et al., 2005; Vollmer, 2008). Under stressful conditions, bacterial cells can activate the peptidoglycan turnover, a phenomenon known as cell wall recycling (Reith and Mayer, 2011). In *Bacillus* species, the predominant fatty acid species of membrane lipids are represented by the branched-chain fatty acids (BCFAs) (de Mendoza et al., 2002; Tojo et al., 2005), where this metabolite represents a precursor (Lowe et al., 1983; Belitsky, 2015). Under adverse states, bacterial cells can adjust BCFA composition to regulate membrane fluidity, allowing survival in a wide range of physical and chemical environments (Singh et al., 2008). Zhu et al. (2014) demonstrated that both *B. subtilis* and *S. aureus* responded to carbon nanotubes (CNTs) toxic stress through changing their fatty acid composition. The modification helped to compensate for the fluidizing effect of nanostructures on the cytoplasmic membrane making it more “rigid” which conforms to the early theory of “homeoviscous adaptation” described by Sinensky (1974). These observations may reflect the signaling around the stress of release of Se atoms and SeNPs from the cytoplasm out of the cell where one can imagine would be disruptive toward cell wall and membrane envelope.

Well after stationary phase (48h), we see the full conversion of ${\mathrm{SeO}}_{3}^{2-}$ to Se^0^ and subsequent increase of SeNP sizes and quantity in the extracellular space (Supplementary Figure 2E). This is accompanied by intracellular accumulation of trans-4-hydroxyproline (Hyp) and L-proline (Pro) (Figures 3E,F), which show osmoprotectant activity (Kim et al., 2017), as well as extracellular accumulation of the amino compound ethanolamine phosphate (Figure 4I). Ethanolamine phosphate is in the phosphatidyl ethanolamine (PE) pathway for this key lipid head group (Kaval and Garsin, 2018). Both of these observations suggest further membrane adaptation and protection likely from the stress of releasing the SeNPs through the cell barrier.

## Conclusion

In the present study, untargeted metabolomics analysis was adopted to explore the effects of the oxyanion ${\mathrm{SeO}}_{3}^{2-}$ on the cells of the Gram-positive bacterium *Bacillus mycoides* SeITE01. This study suggests that this strain faces the toxic effect of ${\mathrm{SeO}}_{3}^{2-}$ by activating several stress defense systems during the growth and through ${\mathrm{SeO}}_{3}^{2-}$ exposure, reduction, and SeNPs production. The identified metabolites were consistent with the hypothesis of intracellular accumulation of osmo-protective solutes and antioxidants, the activation of ROS scavengers, as well as compounds that participate in stabilizing the cytoplasmic membrane. Furthermore, in the cell free spent medium (extracellular), there was a change in metabolites related to oxidative stress, signaling, stress linked amino acids and metabolites involved in modifications of bacterial membranes lipids and cell walls.

## Data Availability Statement

The original contributions presented in the study are included in the article/Supplementary Material, and further inquiries can be directed to the corresponding author.

## Author Contributions

GB, SL, and RT designed the study. GB conducted all the experiments. IL made the metabolomics equipment available. RG analyzed the samples with LC-MS. RC performed the statistical analysis on the datasets. GB, EP, and AP collected the TEM images. GB and RT wrote the manuscript. GV provided his senior authorship by supervising the ultimate reading of the manuscript. All authors approved the submitted version.

## Funding

We acknowledge financial support by the Internationalization award of University of Verona for travel to University of Calgary for data collection. SL was supported by JP2017 grant from the University of Verona. RT and IL recognize the Natural Sciences and Engineering Research council (NSERC) of Canada for Discovery grants supporting the research experiments.

## Conflict of Interest

The authors declare that the research was conducted in the absence of any commercial or financial relationships that could be construed as a potential conflict of interest.

## Publisher’s Note

All claims expressed in this article are solely those of the authors and do not necessarily represent those of their affiliated organizations, or those of the publisher, the editors and the reviewers. Any product that may be evaluated in this article, or claim that may be made by its manufacturer, is not guaranteed or endorsed by the publisher.

## Footnotes

^1^ https://mirror.rcg.sfu.ca/CRAN/

^2^ https://rstudio.com
