# Supplementary material for: Untargeted Metabolomics Investigation on Selenite Reduction to Elemental Selenium by Bacillus mycoides SeITE01
Source: Front Microbiol. 2021 Sep 16;12:711000. doi: 10.3389/fmicb.2021.711000 (PMC8481872; doi:10.3389/fmicb.2021.711000)
Supplement: Supplementary file 1 [file Data_Sheet_1.docx]

Supplementary Material

# Supplementary Figures and Tables

## Supplementary Figures


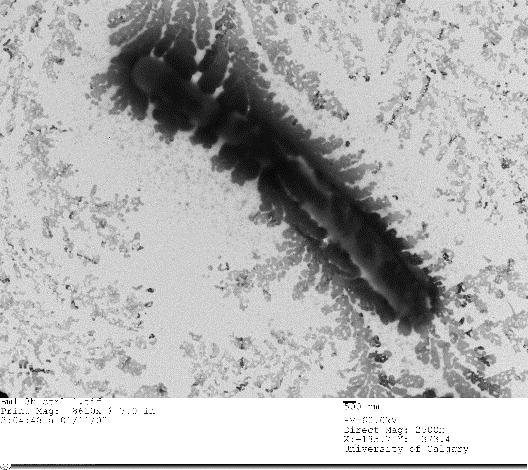


**3h**

2 μm


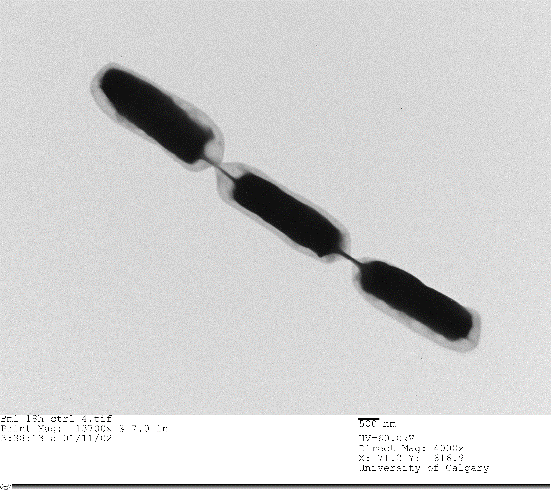


**18h**

2 μm

**12h**


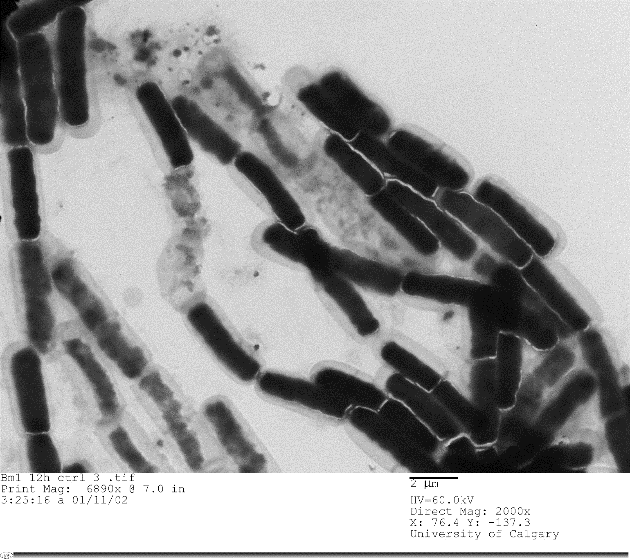


2 μm

**12h**


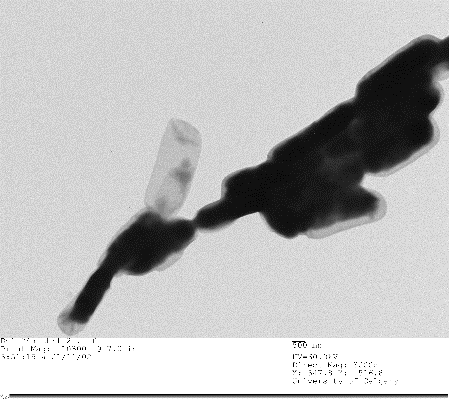


**24h**

2 μm


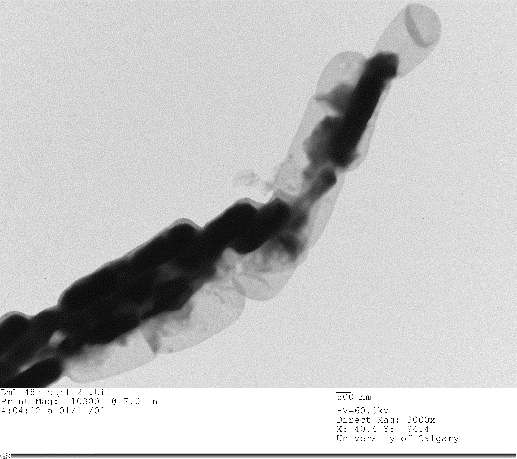


**48h**

2 μm

**(A)**

**(B)**

**(C)**

**(D)**

**(E)**

**Supplementary Figure 1.** TEM time-course of SeITE01 untreated cells grown in NB medium: 3-h (**A**), 12-h (**B**), 18-h (**C**), 24-h (**D**), and 48-h (**E**).


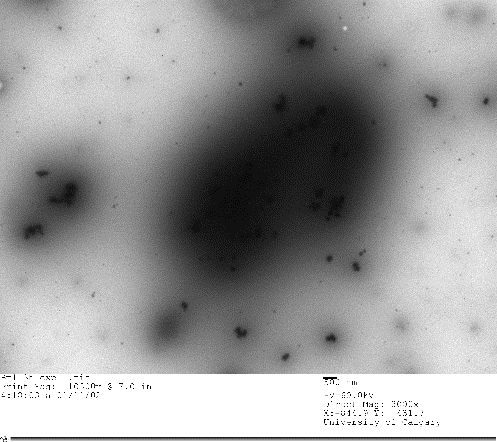


1 μm

**3h**


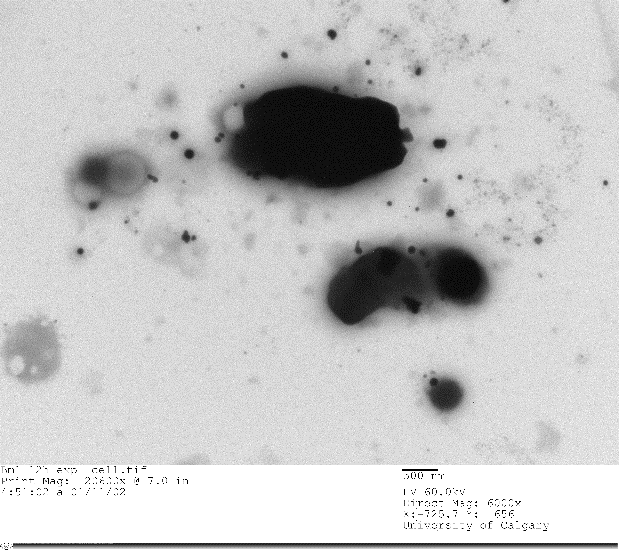


**12h**

1 μm


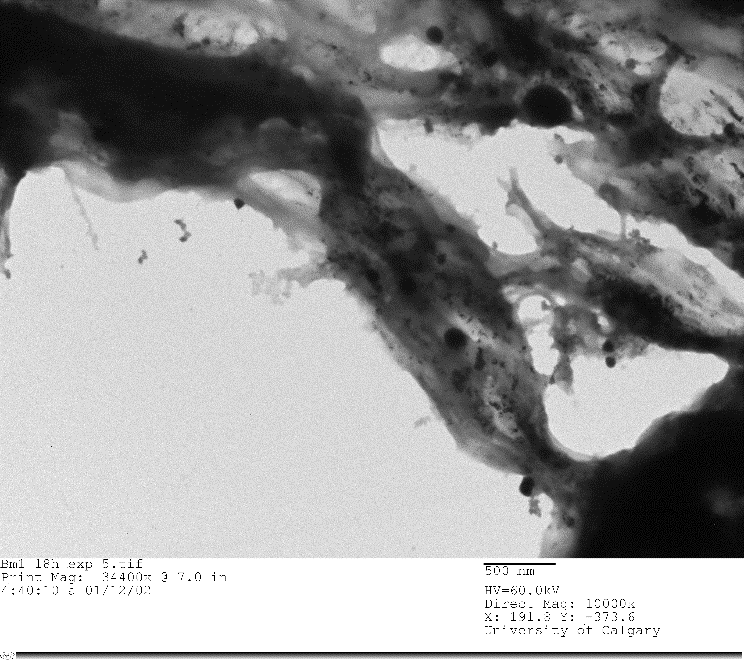


**18h**

500 nm

SeNPs


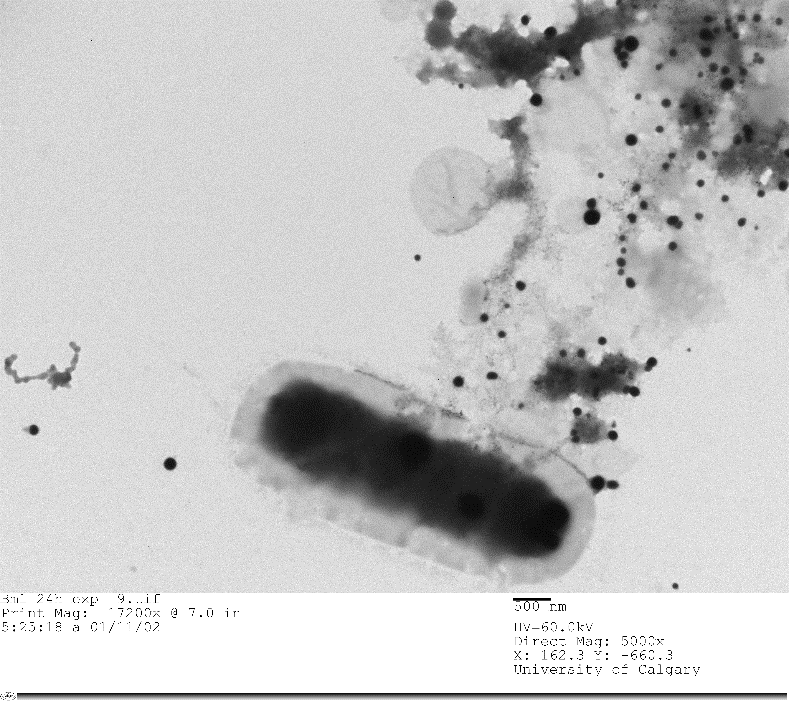


1 μm

**24h**

SeNP

SeNPs

**(A)**

**(B)**

**(C)**

**(D)**

**(E)**

SeNPs


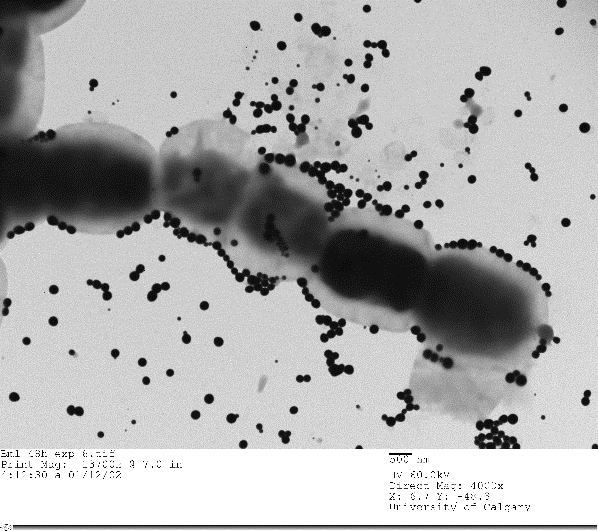


**48h**

1 μm

SeNPs

SeNPs

**Supplementary Figure 2.** TEM time-course of SeITE01 SeO_3_^2-^-treated cells grown in NB medium supplied with 2.0 mM Na_2_SeO_3_: 3-h (**A**), 12-h (**B**), 18-h (**C**), 24-h (**D**), and 48-h (**E**). Black arrows point to biogenic electron-dense selenium nanoparticles (Bio-SeNPs).


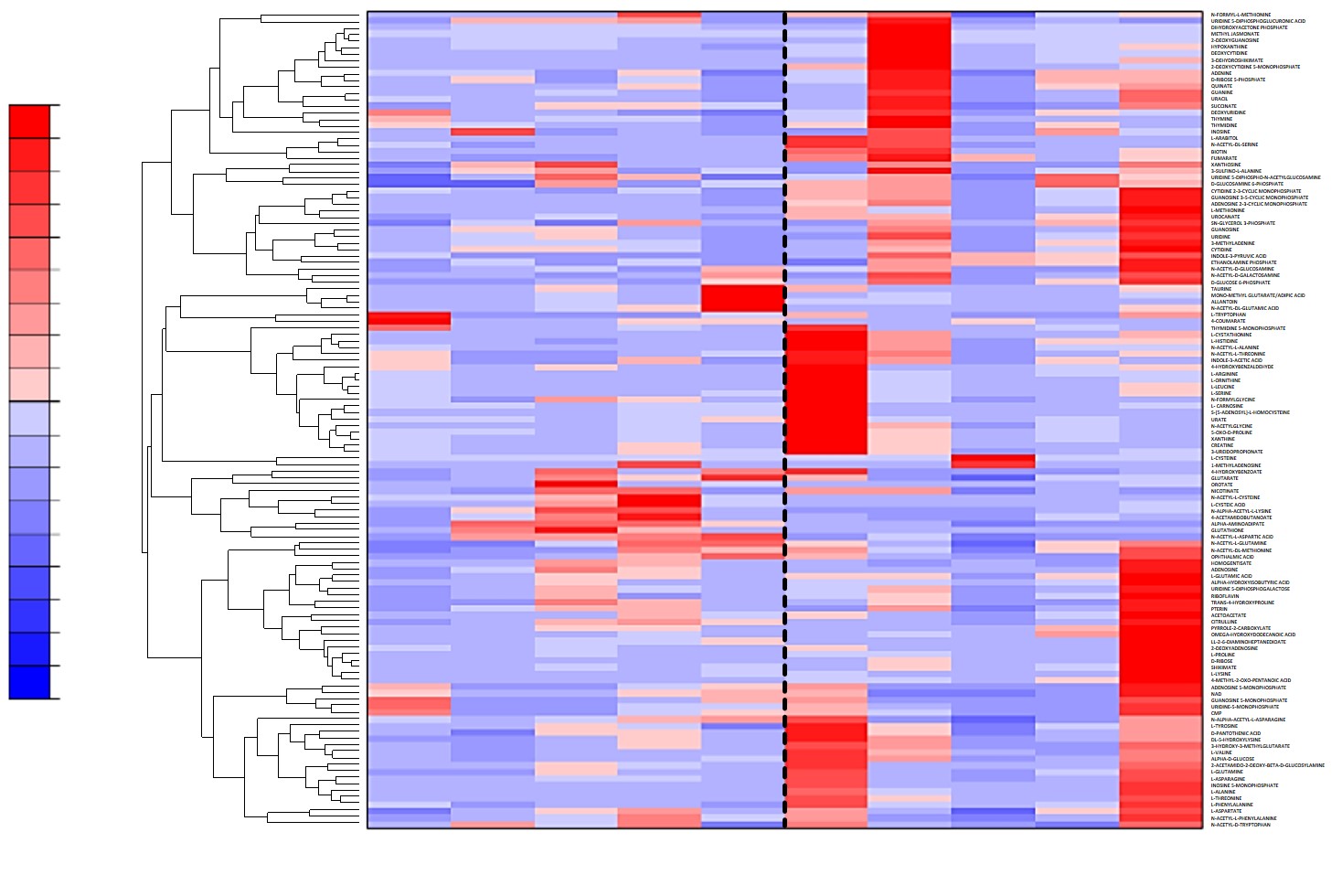


**(A)**

**-2.47**

**2.47**

**Untreated**

**SeO_3_^2-^-treated**

**3-h**

**12-h**

**18-h**

**24-h**

**48-h**

**48-h**

**12-h**

**18-h**

**24-h**

**3-h**


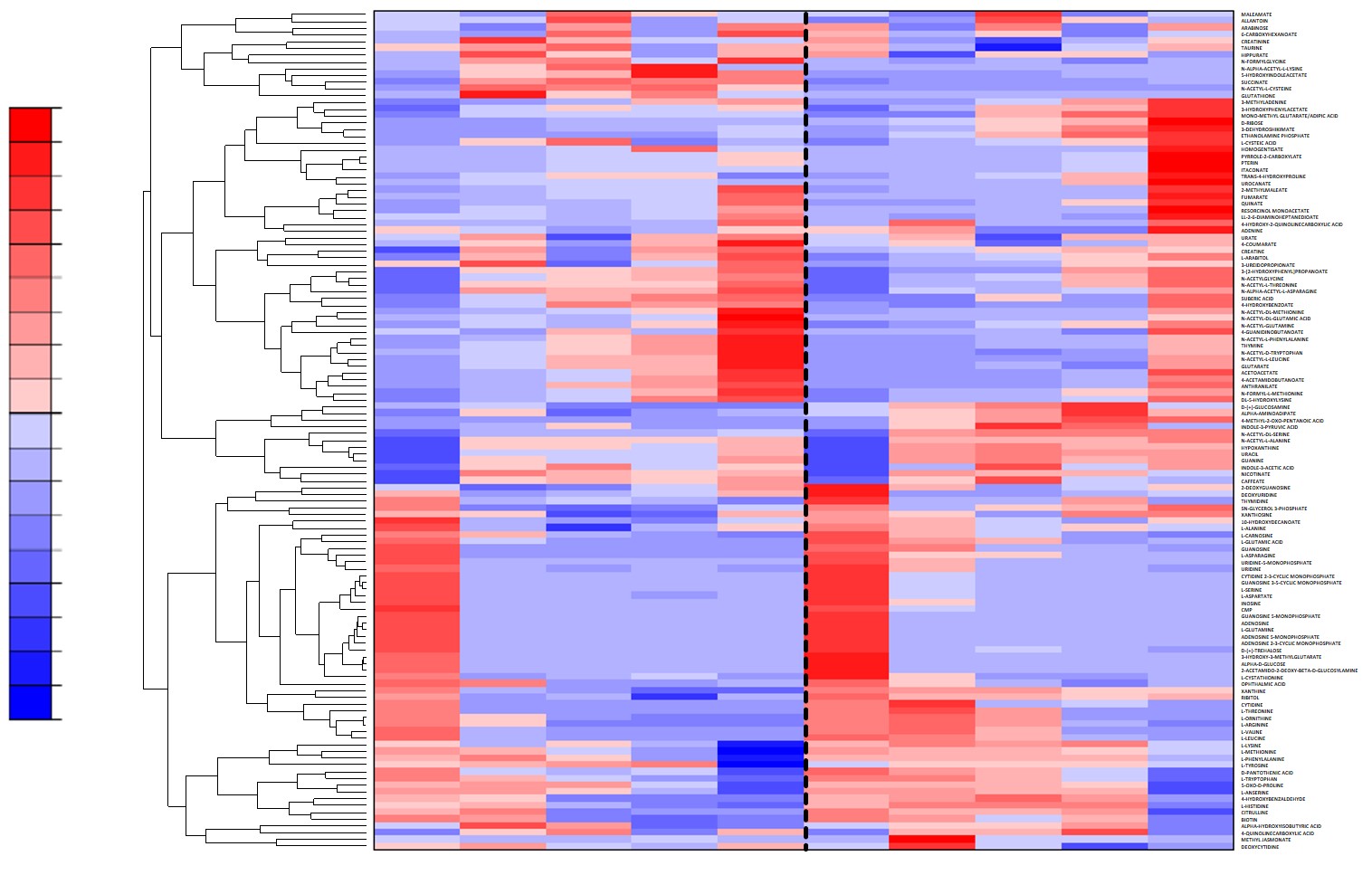


**2.83**

**-2.83**

**3-h**

**12-h**

**18-h**

**24-h**

**48-h**

**3-h**

**12-h**

**18-h**

**24-h**

**48-h**

**SeO_3_^2-^-treated**

**Untreated**

**(B)**

**Supplementary Figure 3.** Clustered heat maps of intracellular (**A**) and extracellular (**B**) compounds of *B. mycoides* SeITE01 recognized after the LC-MS analysis. Rows represent the metabolites, columns the specific time points. For a better reading of the maps, the different treatments were grouped separately to have all the untreated samples on the left and those SeO_3_^2-^-treated on the right. Furthermore, the different color of the squares represent the relative signal intensities of metabolites, where red indicates the most intense and blue the least intense.

(A)

(B)

(C)


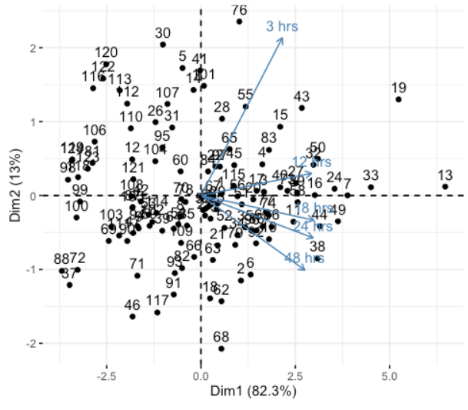

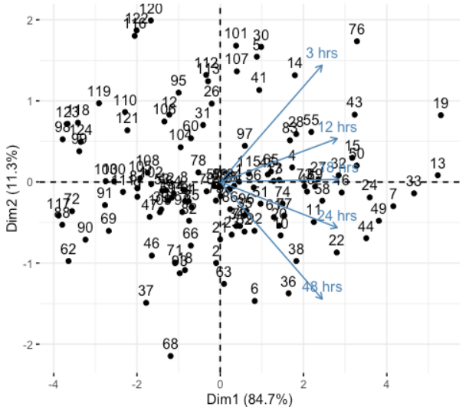

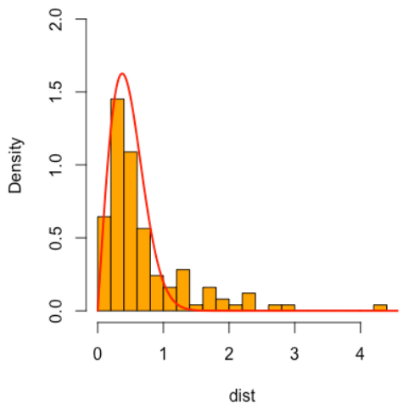

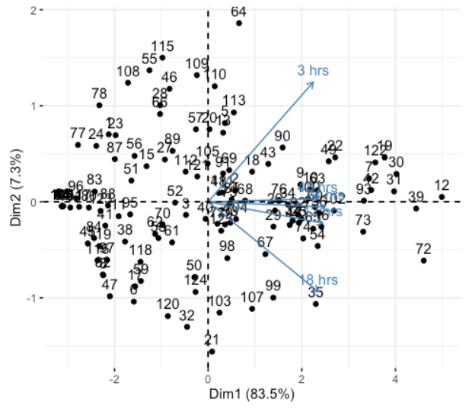

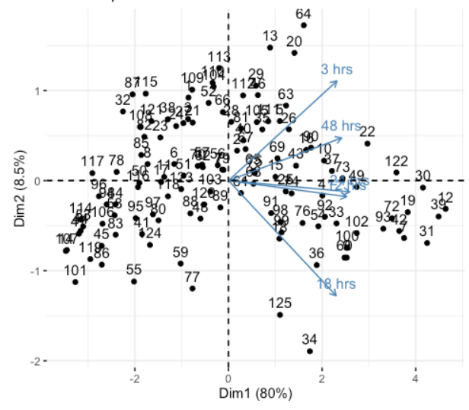

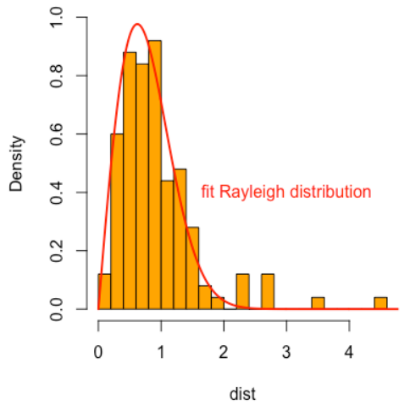


(E)

(D)

(F)

**Supplementary Figure 4.** SeITE01 data processing and statistical elaboration of intracellular (**A-C**) and extracellular (**D-F**) datasets. Panels (**A**) and (**D**) indicate the resulting PCA biplots of the untreated compounds, whist panels (**B**) and (**E**) show the SeO_3_^2-^-treated metabolites. The percentage of variances in the case of the untreated compounds were equal to 83.5% and 80% for the PC1 whilst 7.3% and 8.5% for the PC2. Similar values were also obtained in the case of samples treated with Na_2_SeO_3_, with the PC1 equivalent to 82.3% and 84.7% and the PC2 to 13% and 11.3%. Starting from the temporal information of the PCA, the Euclidean distances between the same metabolites of SeO_3_^2-^-treated and untreated samples were calculated. Panels (**C**) and (**F**) show a new random variable that follows the Rayleigh distribution that it was determined for both the intracellular and extracellular data.

(A)


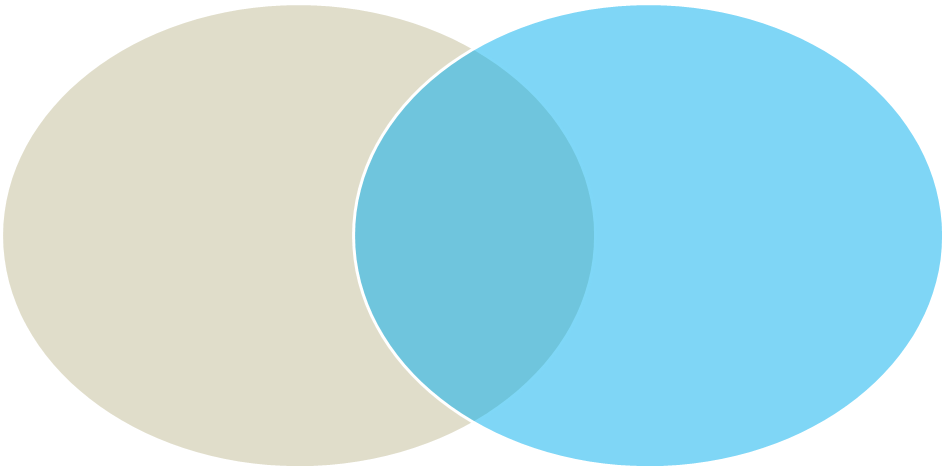


**GUANOSINE**

**GLUTATHIONE**

**4-METHYL-2-OXOPENTANOIC ACID**

**4-ACETAMIDOBUTANOATE**

**N-ACETYL-L-CYSTEINE**

**D-[+]-GLUCOSAMINE**

**L-THREONINE**

**5-HYDROXYINDOLE ACETATE**

**L-ORNITHINE**

LL-2-6-DIAMINOHEPTANEDIOATE

N-α-ACETYL-L-LYSINE

HOMOGENTISATE

ETHANOLAMINE PHOSPHATE

URIDINE

**PCA-SQUARED EUCLIDIAN DISTANCE**

**BAYESIAN INFERENCE-T^2^ STATISTICS**

L-ARGININE

L-ASPARAGINE


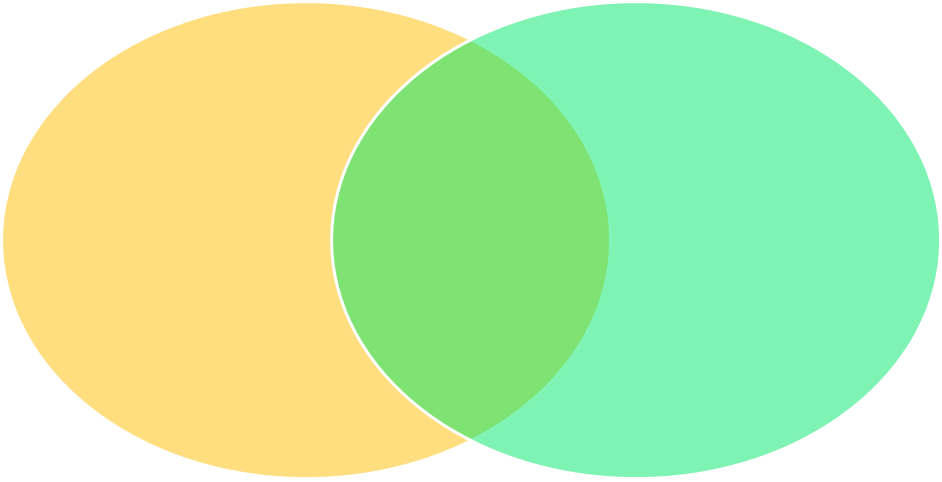


**GLUTATHIONE**

**TRANS-4-HYDROXYPROLINE**

**N-ACETYL-L-CYSTEINE**

L-ASPARTATE

2-ACETAMIDO-2-DEOXY-β-D-GLUCOSYLAMINE

L-ALANINE

INDOLE-3-ACETIC ACID

ADENOSINE 2’, 3’- CYCLIC MONOPHOSPHATE

PYRROLE 2-CARBOXYLATE

INDOLE-3-PYRUVIC ACID

4-HYDROXYBENZOATE

4-COUMARATE

**PCA-SQUARED EUCLIDIAN DISTANCE**

**BAYESIAN INFERENCE-T^2^ STATISTICS**

L-PROLINE

L-METHIONINE

HOMOGENTISATE

2’’-DEOXYGUANOSINE

(B)

**Supplementary Figure 5.** SeITE01 Venn diagrams of intracellular (**A**) and extracellular (**B**) datasets between the two different statistical approaches. The overlap identifies the common metabolites highlighted by the two distinct elaborations.

## Supplementary Tables

**Supplementary Table 1.** SeITE01 intracellular and extracellular statistically relevant metabolites (p-value < 10^-4^) provided by PCA-Squared Euclidian Distance approach.

| INTRACELLULAR METABOLITE | p-value |
| --- | --- |
| Glutathione | 1.2x10^-11^ |
| N-acetyl-N-cysteine | 5.5x10^-8^ |
| 4-hydroxybenzoate | 5.7x10^-5^ |
| Indole-3-pyruvic acid | 8.3x10^-5^ |
| 4-coumarate | 1.5x10^-4^ |
| Trans-4-hydroxyproline | 7.2x10^-4^ |

| **EXTRACELLULAR METABOLITE** | **p-value** | **EXTRACELLULAR METABOLITE** | **p-value** |
| --- | --- | --- | --- |
| N-acetyl-L-cysteine | 10^-14^ | L-threonine | 6.3x10^-7^ |
| Glutathione | 1.8x10^-14^ | Guanosine | 3.5x10^-6^ |
| 4-methyl-2-oxopentanoic acid | 2.5x10^-12^ | L-asparagine | 2x10^-5^ |
| L-arginine | 2.4x10^-9^ | Uridine | 6.4x10^-5^ |
| 5-hydroxyindoleacetate | 5.2x10^-9^ | LL-2-6-diaminoheptanedioate | 8x10^-5^ |
| D-[+]-glucosamine | 1.8x10^-8^ | 4-acetamidobutanoate | 3.9x10^-4^ |
| L-ornithine | 3.9x10^-7^ |  |  |

**Supplementary Table 2.** SeITE01 intracellular and extracellular statistically relevant metabolites occupying the top 10% positions of the ranking list provided by Bayesian Inference-T^2^ statistics approach.

| **INTRACELLULAR METABOLITE** | |
| --- | --- |
| 1) Glutathione | 8) Adenosine 2’, 3’-cyclic monophosphate |
| 2) N-acetyl-L-cysteine | 9) L-aspartate |
| 3) L-alanine | 10) Pyrrol-2-carboxylate |
| 4) 2-acetamido-2-deoxy-β-D-glucosylamine | 11) Homogentisate |
| 5) Trans-4-hydroxyproline | 12) L-methionine |
| 6) Indole-3-acetic acid | 13) 2’’-deoxyguanosine |
| 7) L-proline |  |

| **EXTRACELLULAR METABOLITE** | |
| --- | --- |
| 1) N-acetyl-L-cysteine | 8) L-ornithine |
| 2) D-[+]-glucosamine | 9) N-α-acetyl-L-lysine |
| 3) 4-methyl-2-oxopentanoic acid | 10) Inosine |
| 4) L-threonine | 11) 5-hydroxyindoleacetate |
| 5) Homogentisate | 12) 4-acetamidobutanoate |
| 6) Ethanolamine phosphate | 13) Guanosine |
| 7) Glutathione |  |
